# Gut microbiota and type 1 diabetes: a two-sample bidirectional Mendelian randomization study

**DOI:** 10.3389/fcimb.2023.1163898

**Published:** 2023-05-29

**Authors:** Manjun Luo, Mengting Sun, Tingting Wang, Senmao Zhang, Xinli Song, Xiaoying Liu, Jianhui Wei, Qian Chen, Taowei Zhong, Jiabi Qin

**Affiliations:** ^1^ Department of Epidemiology and Health Statistics, Xiangya School of Public Health, Central South University, Changsha, China; ^2^ Changsha Medical University Public Health Institute, Changsha, China; ^3^ The Hospital of Trade-Business in Hunan Province, Changsha, China; ^4^ Hunan Provincial Key Laboratory of Clinical Epidemiology, Changsha, China

**Keywords:** gut microbiota, T1D, Mendelian randomization, causality, phylum

## Abstract

**Objective:**

The real causal relationship between human gut microbiota and T1D remains unclear and difficult to establish. Herein, we adopted a two-sample bidirectional mendelian randomization (MR) study to evaluate the causality between gut microbiota and T1D.

**Methods:**

We leveraged publicly available genome-wide association study (GWAS) summary data to perform MR analysis. The gut microbiota-related GWAS data from 18,340 individuals from the international consortium MiBioGen were used. The summary statistic data for T1D (n = 264,137) were obtained from the latest release from the FinnGen consortium as the outcome of interest. The selection of instrumental variables conformed strictly to a series of preset inclusion and exclusion criteria. MR-Egger, weighted median, inverse variance weighted (IVW), and weighted mode methods were used to assess the causal association. The Cochran’s Q test, MR-Egger intercept test, and leave-one-out analysis were conducted to identify heterogeneity and pleiotropy.

**Results:**

At the phylum level, only Bacteroidetes was indicated to have causality on T1D (OR = 1.24, 95% CI = 1.01-1.53, *P* = 0.044) in the IVW analysis. When it comes to their subcategories, Bacteroidia class (OR = 1.28, 95% CI = 1.06-1.53, *P* = 0.009, *P*_FDR_ = 0.085), Bacteroidales order (OR = 1.28, 95% CI = 1.06-1.53, *P* = 0.009, *P*_FDR_ = 0.085), and *Eubacterium eligens* group genus (OR = 0.64, 95% CI = 0.50-0.81, *P* = 2.84×10^-4^, *P*_FDR_ = 0.031) were observed to have a causal relationship with T1D in the IVW analysis. No heterogeneity and pleiotropy were detected.

**Conclusions:**

The present study reports that Bacteroidetes phylum, Bacteroidia class, and Bacteroidales order causally increase T1D risk, whereas *Eubacterium eligens* group genus, which belongs to the Firmicutes phylum, causally decreases T1D risk. Nevertheless, future studies are warranted to dissect the underlying mechanisms of specific bacterial taxa’s role in the pathophysiology of T1D.

## 1 Introduction

Type 1 diabetes (T1D) is a chronic, immune-mediated disease characterized by the destruction of pancreatic beta cells and resultant insulin deficiency and hyperglycemia (Dimeglio et al., 2018; Norris et al., 2020). In Europe, the average annual increase in incidence rate was 3%-4% over the past 30 years (Patterson et al., 2009; Patterson et al., 2012; Patterson et al., 2019), which was a little higher than a worldwide estimate (2.8%) ([[NoAuthor]]). This increase can be a direct reflection of the impact of environmental factors on T1D risk. Nowadays, T1D is recognized to result from a complicated intertwinement between environmental factors and microbiome, genome, metabolism, and immune systems, compared with the previous definition of a single autoimmune disorder (Dimeglio et al., 2018). The human microbiome consists of a collection of dynamic microbial communities that colonize in various anatomical organs in the body, among which the gut is the most densely and diversely colonized location (Aggarwal et al., 2022). Recently, a wide association between the human gut microbiota and metabolic disorders (Fan and Pedersen, 2021), cardiovascular diseases (Witkowski et al., 2020), central nervous system disorders (Ma et al., 2019), and autoimmune diseases (Rasouli-Saravani et al., 2023) has earned it growing concern from researchers.

The microbiota of human gut harbors numerous microbes including bacteria, archaea, eukarya, viruses, and parasites, with bacteria as the dominant population (Lozupone et al., 2012). Specifically, there are four predominant bacterial phyla, namely, Bacteroidetes, Firmicutes, Proteobacteria, and Actinobacteria, the first two of which constitute over 90% of the gut microbiota (Zoetendal et al., 2008; Arumugam et al., 2011; Faith et al., 2013). Previous studies revealed a significantly decreased bacterial diversity including both alpha diversity and Shannon diversity index in T1D progressors in the time window between seroconversion and clinical T1D (Giongo et al., 2011; Kostic et al., 2015; Knip and Siljander, 2016). This decrease occurring before disease diagnosis indicated that the altered gut microbiota might contribute to the initiation or development of T1D. In addition, existing data reported that microbiome composition in the gut differed significantly between T1D individuals and healthy control subjects (Murri et al., 2013; Leiva-Gea et al., 2018). Exactly, T1D cases showed a significant increase in the abundance of Bacteroidetes and a significant decrease in the abundance of Firmicutes, Actinobacteria, and Proteobacteria with respect to controls. This T1D-related gut dysbiosis could be characterized by increased lipopolysaccharide (LPS) biosynthesis and decreased butyrate production, and the two metabolites had been evidenced in T1D mice to exert destructive and protective effects, respectively, on glucose metabolism and islet structure and function (Yuan et al., 2022). An experimental study recently performed a series of fecal oral transplants using non-obese diabetic (NOD) and resistant (NOR) mice and demonstrated that NOR mice transplanted with microbiota from NOD displayed greater insulitis compared with non-transplanted NOR mice (Brown et al., 2016). Briefly, a growing number of scientific discoveries lend increasing support to the notion that alterations in the gut microbiota may have a causal relation with T1D risk. Nevertheless, existing studies exhibit limitations including insufficient statistical robustness attributed to the limited sample size, inherent defects of observational studies, and the gap between human research and animal research, leaving the real causal relation between gut microbiota and T1D unclear and waiting to be elucidated.

Mendelian randomization (MR) utilizes genetic variants which are robustly associated with the exposure as instrumental variables (IVs) to infer causality between a risk factor and a healthy outcome (Davey Smith and Hemani, 2014; Bowden and Holmes, 2019). The approach provides stronger evidence for causal inference than observational epidemiology since it can largely overcome the influence on estimated associations of potential confounding, reverse causation, and various other sources of bias (Smith and Ebrahim, 2004; Zuccolo and Holmes, 2017). A newly published research performed a series of genome-wide association studies (GWASs) to explore the effect of host genetic loci on the abundance of various intestinal bacterial taxa and provided the summary statistics for 211 taxa (Kurilshikov et al., 2021). This made it feasible to determine the potential causal effects of human gut microbiota on different disease outcomes using the MR approach. To date, there is an MR research which has explored the causal relationship of gut microbiota and T1D before (Xu et al., 2021), but it only analyzed 131 bacterial taxa at the genus level. In the current study, we aimed to provide more comprehensive information about the causality between gut microbiota and T1D by including different levels (phylum, class, order, family, and genus) of bacterial taxa and leveraging updated GWAS summary data for T1D with a larger sample size. Meanwhile, to exclude the possibility that T1D has a causal effect on gut microbiota, MR analyses were also performed in the reverse direction.

## 2 Methods

### 2.1 Data sources for gut microbiota and T1D

The gut microbiota-related GWAS data were obtained from the international consortium MiBioGen (Wang et al., 2018), which conducted a large-scale, multiethnic, genome-wide meta-analysis of the associations between autosomal human genetic variants and the gut microbiome (Brown et al., 2016). The meta-analysis covered 18,340 participants from 24 cohorts from the USA, Canada, Israel, South Korea, Germany, Denmark, the Netherlands, Belgium, Sweden, Finland, and the UK. After adjustment for age, sex, technical covariates, and genetic principal components, the results from the quantitative microbiome trait loci (mbQTL) analysis in the study produced 211 microbial taxa-related GWAS summary statistics, including 9 phyla, 16 classes, 20 orders, 35 families (with 3 unknown families), and 131 genera (with 12 unknown genera). More details about the microbiota data could be found in an original study (Brown et al., 2016). In our study, we mainly focused on four dominant phyla, namely, Actinobacteria, Bacteroidetes, Firmicutes, and Proteobacteria, and their subcategories considering their wide distribution in the human gut and existing research foundation. Therefore, we utilized the Taxonomy Browser tool in NCBI (https://www.ncbi.nlm.nih.gov/guide/taxonomy/) to consult the taxonomy tree for every microbial taxon. Among 211 taxa, there are 2 classes, 3 orders, 3 families, and 10 genera which belonged to Actinobacteria; 1 class, 1 order, 5 families, and 12 genera which belonged to Bacteroidetes; 4 classes, 5 orders, 14 families, and 87 genera which belonged to Firmicutes; and 4 classes, 5 orders, 6 families, and 7 genera which belonged to Proteobacteria. The detailed categorization can be seen in **Supplementary Table 1**. In addition, the rest of bacterial taxa, namely, the other five phyla and their subcategories, were also included in the MR analyses to provide potentially extra evidence for causality. Consequently, after excluding 15 unknown families and genera, a total of 196 taxa at different levels were selected as the exposure of interest in our study. The summary statistics data of T1D (case: 8,671, control: 255,466), adjusted for sex, age, genotyping batch, and the first 10 genetic principal components, were obtained from the latest release from the FinnGen consortium in June 2022 (Kurki et al., 2022). The study needs no additional ethical approval since the original studies have received appropriate ethics and institutional review board approval.

### 2.2 The assumptions of an MR study

**Figure 1:**
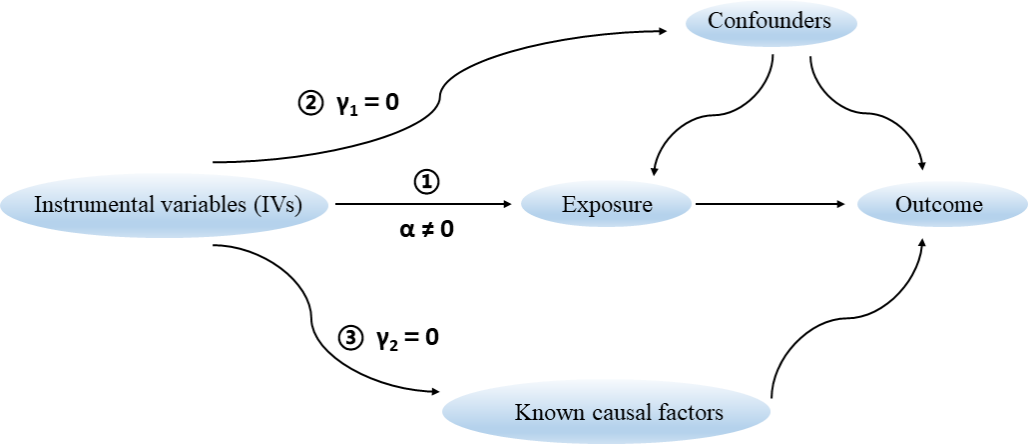
Three key assumptions for a valid Mendelian randomization study.

There are three key assumptions that hold for a valid MR study (**Figure 1**) (Davies et al., 2018). Firstly, the selected genetic variants as IVs are robustly associated with the exposure of interest. The F statistic is generally used to estimate the strength of instruments. It can be calculated *via* the formula F = R^2^(n-k-1)/k(1-R^2^), where R^2^ represents the proportion of variance explained by instruments, n represents the sample size, and k is the number of selected IVs. We set a conventional threshold value of F statistic > 10 to avoid potential weak instrument bias in this study (Burgess et al., 2013). Secondly, there are no unmeasured confounders of the associations between genetic variants and outcome. Thirdly, the genetic variants affect the outcome only through their effect on the exposure of interest, that is, there is no horizontal pleiotropy between genetic variants and outcome.

### 2.3 Inclusion and exclusion of IVs

**Figure 2:**
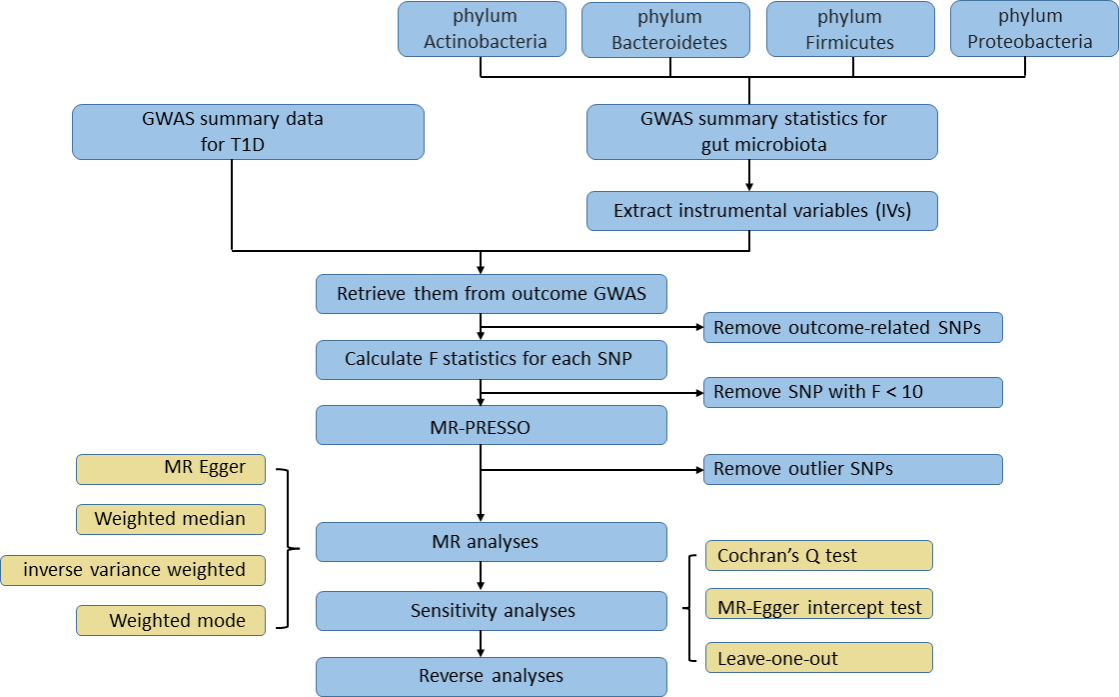
The flowchart of the Mendelian randomization study revealing the causal relationship between gut microbiota and T1D.

To identify the causal effect from human gut microbiota on T1D, we selected SNPs that showed an association at *P* < 1×10^-5^ as instruments in our MR analysis. This value was identified as the optimal threshold in many gut microbiota-related MR research to maximize the amount of genetic variance explained by the genetic predictors and increase the number of eligible SNPs in order to perform sensitivity analysis (Brown et al., 2016; Sanna et al., 2019). Then, independent SNPs were selected based on linkage disequilibrium [LD] r^2^ < 0.001 in a 10,000-kb window in 1000G EUR data using the clumping procedure within the TwoSampleMR package. If no shared SNP existed among GWAS data of exposure and outcome, a proxy SNP with r^2^ > 0.8 would replace it. After IVs were retrieved from the T1D GWAS data, we then removed the SNP that was significantly associated with TID based on the following criterion: *P*_outcome_ < *P*_exposure_. F statistics were calculated for each SNP after the harmonization process; an SNP with F statistics less than 10 would be considered as a weak instrument and be excluded. MR pleiotropy residual sum and outlier (MR‐PRESSO) tests, which are optimally applicable when a horizontal pleiotropy is found in less than 50% of the instruments, were performed to detect and remove outlier instruments (Verbanck et al., 2018). Only SNPs passing the stringent filtering procedure could they access the subsequent MR analysis. To test if T1D affected the microbiome composition, we selected genetic variants associated with T1D at the genome-wide significance level (*P* < 5×10^−8^) as instruments in reverse MR analysis. The rest of filtering steps were the same as the former one. The study flowchart is presented in **Figure 2**.

### 2.4 Mendelian randomization analysis

We performed the MR analysis using four different approaches [MR-Egger, weighted median, random-effect inverse variance weighted (IVW), weighted mode] to calculate causal estimates between gut microbiota composition and the risk of T1D. MR-Egger can give a consistent causal effect estimate even when all the genetic variants have a pleiotropic effect as long as the association of each genetic variant with the exposure is independent of the pleiotropic effect (Bowden et al., 2015). Weighted median requires that at least 50% of the weight in the analysis stems from variants that are valid instruments (Bowden et al., 2016). IVW is essentially a meta-analysis method based on the assumption that instruments can affect the outcome only through the exposure of interest and not by any alternative pathway (Bowden et al., 2017). Weighted mode is consistent when the largest subset of instruments which identify the same causal effect are valid instruments, even if the majority of others are invalid (Hartwig et al., 2017). Thus, a causal relationship was considered when a significant *P* value (*P* < 0.05) derived from any of these four methods in the MR analysis was detected.

Sensitivity analysis had also been performed for significant estimates to detect potential heterogeneity and pleiotropy. The Cochran’s Q test was used to identify heterogeneity. The MR-Egger intercept test was conducted to assess horizontal pleiotropy. An insignificant *P* value (*P* > 0.05) was defined as the absence of heterogeneity or pleiotropy. In addition, we also applied the leave-one-out analyses to check whether the causal estimates could be biased or driven by a single SNP through removing each instrumental SNP in turn to repeat the IVW analyses.

All the analyses were completed with R (version 4.2.1). R package “TwoSampleMR” (version 0.5.6) and “stats” (version 4.2.1) were utilized. At the phylum-level test, significant results were those with the *P* values < 0.05. When considering the causal relationship between subcategories of each phylum and T1D risk, a false discovery rate *P* value (*P*_FDR_), calculated based on the Benjamini–Hochberg (BH) method, was used to adjust for multiple testing. Significant results were those with the *P*_FDR_ < 0.1; meanwhile *P <*0.05 but *P*_FDR_ > 0.1 was considered as nominally significant.

## 3 Results

### 3.1 Causal association of four dominant phyla and their subcategories with T1D

At the phylum level, 15 index SNPs were selected to genetically predict Actinobacteria, 10 index SNPs were selected to genetically predict Bacteroidetes, 14 index SNPs were selected to genetically predict Firmicutes, and 12 index SNPs were selected to genetically predict Proteobacteria. The F statistics were calculated for each SNP and were all larger than the threshold value of 10, indicating strong instruments (Pierce et al., 2011). Among four phyla, only Bacteroidetes was indicated to have causality on T1D (OR = 1.24, 95% CI = 1.01-1.53, *P* = 0.044) in the IVW analysis.

Subsequently, 2 classes, 3 orders, 3 families, and 10 genera of Actinobacteria; 1 class, 1 order, 5 families, and 12 genera of Bacteroidetes; 4 classes, 5 orders, 14 families, and 87 genera of Firmicutes; and 4 classes, 5 orders, 6 families, and 7 genera of Proteobacteria were included in the MR analysis to further explore the causal relationship between gut microbiota and T1D. We identified several significant and nominally significant taxa, which had causal effects on T1D, and these taxa mainly belonged to Bacteroidetes and Firmicutes (**Tables 1**, **2**). The full analysis results are provided in **Supplementary Tables 2–5**.

**Table 1:** Significant and nominally significant Mendelian randomization estimates of the associations from Bacteroidetes on T1D.

Taxa	Gut microbiota (exposure)	Trait (outcome)	Nsnp	Methods	Beta	SE	OR (95%CI)	*P* value	*P*_FDR_	Heterogeneity		Horizontal pleiotropy		
										Cochran’s Q	*P* value	Egger intercept	SE	*P* value
Phylum	Bacteroidetes	T1D	10	MR-Egger	-0.28	0.29	0.75 (0.43-1.32)	0.352	0.352	6.344	0.705	0.036	0.019	0.099
				Weighted median	0.21	0.15	1.24 (0.94-1.64)	0.146	0.146					
				Inverse variance weighted	0.21	0.11	1.24 (1.01-1.53)	**0.044**	**0.044**					
				Weighted mode	0.24	0.23	1.27 (0.82-1.97)	0.322	0.322					
Class	Bacteroidia	T1D	13	MR-Egger	-0.11	0.26	0.90 (0.54-1.50)	0.682	0.864	6.528	0.887	0.025	0.017	0.176
				Weighted median	0.22	0.13	1.25 (0.97-1.60)	0.084	0.488					
				Inverse variance weighted	0.24	0.09	1.28 (1.06-1.53)	**0.009**	**0.085**					
				Weighted mode	0.22	0.19	1.24 (0.84-1.84)	0.276	0.926					
Order	Bacteroidales	T1D	13	MR-Egger	-0.11	0.26	0.90 (0.54-1.50)	0.682	0.864	6.528	0.887	0.025	0.017	0.176
				Weighted median	0.22	0.12	1.25 (0.98-1.59)	0.067	0.488					
				Inverse variance weighted	0.24	0.09	1.28 (1.06-1.53)	**0.009**	**0.085**					
				Weighted mode	0.22	0.20	1.24 (0.85-1.82)	0.302	0.926					
Family	Prevotellaceae	T1D	15	MR-Egger	-0.64	0.26	0.53 (0.31-0.88)	**0.029**	0.275	18.400	0.189	0.044	0.018	0.032
				Weighted median	-0.07	0.11	0.93 (0.76-1.15)	0.546	0.909					
				Inverse variance weighted	-0.04	0.08	0.96 (0.82-1.14)	0.654	0.859					
				Weighted mode	-0.01	0.18	0.99 (0.68-1.45)	0.968	0.985					
Family	Rikenellaceae	T1D	16	MR-Egger	0.67	0.23	1.95 (1.23-3.08)	**0.013**	0.247	20.157	0.166	-0.045	0.017	0.016
				Weighted median	-0.02	0.11	0.98 (0.78-1.24)	0.884	0.909					
				Inverse variance weighted	0.07	0.09	1.07 (0.90-1.27)	0.458	0.859					
				Weighted mode	-0.10	0.24	0.91 (0.59-1.40)	0.682	0.926					

Nsnp, number of snps.

Significant estimate is defined as *P*_FDR_ <0.1; nominal significant estimate is defined as *P* value <0.05.

Cochran’s Q-derived *P* value and MR-Egger intercept-derived *P* value < 0.05 is significant.

**Table 2:** Significant and nominally significant Mendelian randomization estimates of the associations from Firmicutes on T1D.

Taxa	Gut microbiota (exposure)	Trait (outcome)	Nsnp	Methods	Beta	SE	OR(95%CI)	*P* value	*P*_FDR_	Heterogeneity		Horizontal pleiotropy			
										Cochran’s Q	*P* value		Egger intercept	SE	*P* value
Class	Clostridia	T1D	10	MR-Egger	-0.38	0.41	0.68(0.30-1.54)	0.385	0.972	7.819	0.552		0.013	0.027	0.650
				Weighted median	-0.23	0.13	0.79(0.61-1.03)	0.081	0.856						
				Inverse variance weighted	-0.19	0.10	0.83(0.68-1.00)	**0.049**	0.588						
				Weighted mode	-0.22	0.20	0.80(0.54-1.18)	0.285	0.953						
Family	Family XI	T1D	8	MR-Egger	0.35	0.30	1.42(0.79-2.55)	0.283	0.972	8.439	0.295		-0.066	0.039	0.147
				Weighted median	-0.19	0.07	0.82(0.71-0.95)	**0.004**	0.302						
				Inverse variance weighted	-0.14	0.05	0.87(0.79-0.96)	**0.007**	0.378						
				Weighted mode	-0.25	0.14	0.78(0.60-1.02)	0.111	0.953						
Family	Peptococcaceae	T1D	8	MR-Egger	-0.14	0.29	0.87(0.50-1.52)	0.634	0.988	9.908	0.194		-0.005	0.025	0.835
				Weighted median	-0.19	0.11	0.83(0.66-1.04)	0.102	0.856						
				Inverse variance weighted	-0.20	0.09	0.82(0.68-0.98)	**0.034**	0.588						
				Weighted mode	-0.22	0.17	0.80(0.58-1.11)	0.222	0.953						
Family	Veillonellaceae	T1D	19	MR-Egger	-0.01	0.12	0.99(0.78-1.26)	0.923	0.988	16.211	0.578		0.013	0.010	0.187
				Weighted median	0.08	0.09	1.09(0.91-1.30)	0.360	0.858						
				Inverse variance weighted	0.13	0.06	1.14(1.02-1.29)	**0.027**	0.588						
				Weighted mode	0.00	0.13	1.00(0.79-1.28)	0.980	0.998						
Genus	*Butyricicoccus*	T1D	8	MR-Egger	0.15	0.23	1.16(0.74-1.82)	0.552	0.988	9.059	0.248		0.008	0.020	0.705
				Weighted median	0.12	0.14	1.12(0.86-1.47)	0.397	0.858						
				Inverse variance weighted	0.22	0.11	1.25(1.01-1.55)	**0.041**	0.588						
				Weighted mode	0.11	0.15	1.11(0.83-1.48)	0.492	0.953						
Genus	*Dorea*	T1D	10	MR-Egger	-0.38	0.30	0.69(0.38-1.24)	0.251	0.972	9.558	0.387		0.012	0.020	0.569
				Weighted median	-0.34	0.15	0.71(0.53-0.95)	**0.020**	0.540						
				Inverse variance weighted	-0.21	0.10	0.81(0.66-1.00)	**0.048**	0.588						
				Weighted mode	-0.44	0.25	0.64(0.39-1.05)	0.110	0.953						
Genus	*Eubacterium eligens* group	T1D	6	MR-Egger	-0.63	0.51	0.53(0.20-1.44)	0.282	0.972	5.572	0.350		0.015	0.040	0.728
				Weighted median	-0.44	0.16	0.64(0.47-0.88)	**0.006**	0.302						
				Inverse variance weighted	-0.45	0.12	0.64(0.50-0.81)	**0.000**	**0.031**						
				Weighted mode	-0.56	0.27	0.57(0.34-0.97)	0.093	0.953						
Genus	*Holdemania*	T1D	14	MR-Egger	0.40	0.18	1.50(1.05-2.12)	**0.044**	0.972	16.392	0.229		-0.038	0.017	**0.047**
				Weighted median	-0.03	0.09	0.97(0.82-1.15)	0.763	0.896						
				Inverse variance weighted	0.03	0.07	1.03(0.90-1.18)	0.671	0.978						
				Weighted mode	-0.18	0.19	0.84(0.56-1.26)	0.383	0.953						
Genus	*Lachnospiraceae* UCG008	T1D	11	MR-Egger	-0.29	0.36	0.75(0.37-1.51)	0.437	0.972	13.119	0.217		0.014	0.037	0.708
				Weighted median	-0.12	0.08	0.89(0.76-1.05)	0.165	0.858						
				Inverse variance weighted	-0.15	0.07	0.86(0.75-0.97)	**0.019**	0.588						
				Weighted mode	-0.11	0.13	0.89(0.68-1.17)	0.410	0.953						
Genus	*Ruminococcaceae* UCG010	T1D	6	MR-Egger	-0.27	0.28	0.76(0.44-1.31)	0.382	0.972	1.083	0.956		0.005	0.020	0.821
				Weighted median	-0.21	0.13	0.81(0.63-1.04)	0.097	0.856						
				Inverse variance weighted	-0.21	0.10	0.81(0.66-0.99)	**0.038**	0.588						
				Weighted mode	-0.22	0.16	0.80(0.58-1.11)	0.236	0.953						
Genus	*Ruminococcus2*	T1D	15	MR-Egger	0.22	0.25	1.24(0.76-2.04)	0.407	0.972	33.898	**0.002**		-0.016	0.020	0.432
				Weighted median	0.19	0.11	1.22(0.98-1.51)	0.064	0.856						
				Inverse variance weighted	0.03	0.10	1.03(0.84-1.26)	0.769	0.986						
				Weighted mode	0.25	0.11	1.29(1.02-1.63)	**0.038**	0.953						
Genus	*Veillonella*	T1D	5	MR-Egger	-2.14	0.81	0.12(0.02-0.57)	0.077	0.972	9.763	**0.045**		0.180	0.062	0.063
				Weighted median	0.38	0.15	1.46(1.08(1.98)	**0.010**	0.360						
				Inverse variance weighted	0.16	0.16	1.18(0.86-1.62)	0.313	0.835						
				Weighted mode	0.39	0.18	1.47(1.01-2.15)	0.100	0.953						

Nsnp, the number of snps.

Significant estimate is defined as *P*_FDR_ <0.1; nominal significant estimate is defined as *P* value <0.05.

Cochran’s Q-derived *P* value and MR-Egger intercept-derived *P* value < 0.05 is significant.

For Actinobacteria (**Supplementary Table 2**), only one taxa, named *Olsenella* (genus), showed a nominally significant correlation with T1D (OR = 1.10, 95% CI = 1.01-1.20, *P* = 0.027, *P*_FDR_ = 0.486) in the IVW analysis. Nevertheless, the reverse MR analysis failed to exclude the possibility that T1D is causally associated with *Olsenella*.

For Bacteroidetes (**Table 1**; **Supplementary Table 3**), totally four taxa were identified to have causality on T1D, namely, two significant taxa, Bacteroidia (class) and Bacteroidales (order), and two nominally significant taxa, Prevotellaceae (family) and Rikenellaceae (family). We observed that Bacteroidia (OR = 1.28, 95% CI = 1.06-1.53, *P* = 0.009, *P*_FDR_ = 0.085) and Bacteroidales (OR = 1.28, 95% CI = 1.06-1.53, *P* = 0.009, *P*_FDR_ = 0.085) had a causal relationship with T1D in the IVW analysis; meanwhile, no heterogeneity or pleiotropy was detected. When it comes to the family level, nominal significance was found in Prevotellaceae (OR = 0.53, 95% CI = 0.31-0.88, *P* = 0.029, *P*_FDR_ = 0.275) and Rikenellaceae (OR = 1.95, 95% CI = 1.23-3.08, *P* = 0.013, *P*_FDR_ = 0.247) using the MR-Egger method. However, both *P*-values of MR-Egger intercept tests were <0.05, indicating that there was horizontal pleiotropy observed.

For Firmicutes (**Table 2**; **Supplementary Table 4**), totally 12 taxa were identified to have a causal effect on T1D, including 1 significant taxa named *Eubacterium eligens* group (genus) and 11 nominally significant taxa, which included Clostridia (class), Family XI (family), Peptococcaceae (family), Veillonellaceae (family), *Butyricicoccus* (genus), *Dorea* (genus), *Holdemania* (genus), *Lachnospiraceae* UCG008 (genus), *Ruminococcaceae* UCG010 (genus), *Ruminococcus2* (genus), and *Veillonella* (genus). The genetically predicted *Eubacterium eligens* group was significantly associated with decreased risk of T1D (OR = 0.64, 95% CI = 0.50-0.81, *P* = 2.84×10^-4^, *P*_FDR_ = 0.031) in the IVW analysis, with no detected heterogeneity or pleiotropy. Among 11 nominally significant taxa, heterogeneity was observed with the Cochran Q-derived *P*-values < 0.05 for Ruminococcus2 and Veillonella, and pleiotropy was found with the MR-Egger intercept test-derived *P*-value < 0.05 for Holdemania. The detailed results for the rest of eight nominally significant taxa are represented in **Table 2**.

**Figure 3:**
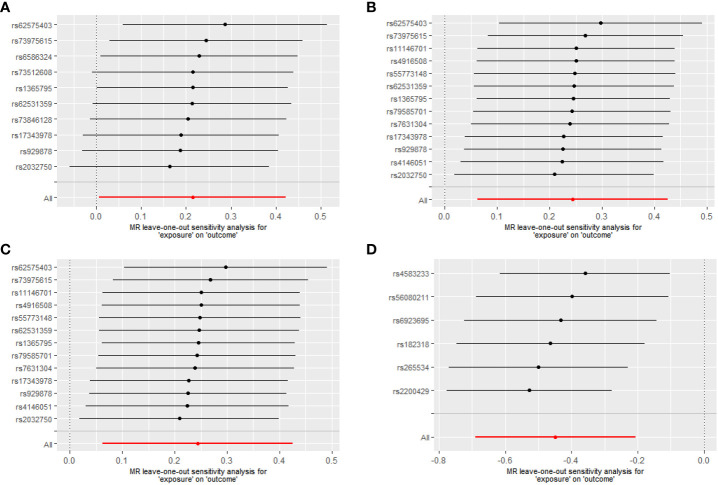
Leave-one-out analysis of the causal effects of the significant microbial taxa on T1D **(A)** Causal effect of phylum. Bacteroidetes on T1D **(B)** Causal effect of class. Bacteroidia on T1D **(C)** Causal effect of order. Bacteroidales on T1D **(D)** Causal effect of genus. *Eubacterium eligens* group on T1D.

For Proteobacteria (**Supplementary Table 5**), neither significant nor nominally significant taxa were found in the MR analysis. **Figure 3** summarizes the results of the causal effect from four significant taxa on T1D in the leave-one-out analysis.

### 3.2 Causal association of the other five phyla and their subcategories with T1D

To obtain more extensive information on the causality between human gut microbiota and T1D, we also included the less abundant bacterial taxa (totally 23) at different levels in the MR analyses. The results are summarized in **Supplementary Table 6**. Among the 23 taxa, totally three were found to be causally associated with T1D. Specifically, Tenericutes (phylum) showed a significant correlation with T1D (OR = 0.80, 95%CI = 0.64-0.99, *P* = 0.037); nominal significance was observed in class Mollicutes (OR = 0.80, 95%CI = 0.64-1.00, *P* = 0.046, *P*_FDR_ = 0.138) and order NB1n (OR = 0.58, 95%CI = 0.37-0.90, *P* = 0.036, *P*_FDR_ = 0.108). However, significant heterogeneity or horizontal pleiotropy was detected in the three taxa since the Cochran Q-derived *P*-values < 0.05 or the MR-Egger intercept test-derived *P*-value < 0.05.

## 4 Discussion

The role of gut microbiota has been implicated in the occurrence or development of T1D from a multiple-evidence chain in previous studies. Nonetheless, the real causal relationship between human gut microbiota and T1D remains difficult to establish ascribing to intrinsic defects of the observational study and potential ethic issues limiting the experimental study conducted in human beings. Thus, our study aimed to assess the causal relation of genetically predicted gut microbiota and T1D using the two-sample bidirectional MR method. The results of our study suggested that Bacteroidetes phylum, Bacteroidia class, and Bacteroidales order significantly increased the risk of T1D, whereas the *Eubacterium eligens* group genus, belonging to the Firmicutes phylum, significantly decreased the risk of T1D. The findings in the current study allow for gut microbiota being not only a potential indicator for the earlier identification of higher-risk individuals of T1D but also a breakthrough point for achieving more optimized preventive and treatment strategies.

Interestingly, a previous MR study, with the aim of evaluating the causal association between gut microbiota and autoimmune diseases (ADs), revealed that a higher relative abundance of the Bifidobacterium genus was associated with a higher risk of T1D. Our study reported quite different results; this discrepancy may be attributed to a more relaxed threshold of *P* value (1×10^-5^ rather than 5×10^-8^) for the selection of IVs and a larger sample size for the T1D GWAS data used. The value we used was identified as the optimal threshold for selection of genetic predictors associated with the gut microbiome since it led to a larger variance explained and it had been applied in many gut microbiota-related MR studies (Brown et al., 2016; Sanna et al., 2019). Currently, the available literatures mainly focused on human gut microbiota at the phylum level and genus level to determine the compositional changes between diabetic or prediabetic individuals and healthy controls. Studies evaluating the associations between the abundance of Actinobacteria and Proteobacteria and T1D exhibited inconsistent results, with positive, negative, and null associations all having been reported (Brown et al., 2011; Murri et al., 2013; Davis-Richardson et al., 2014; Leiva-Gea et al., 2018). Our study did not identify any significant taxa in the two phyla and their descendants to have a causal effect on T1D. As for the Bacteroidetes phylum, varied studies reported unexpectedly consistent results that the Bacteroidetes abundance increased significantly either in T1D progressors over time or in T1D cases relative to controls, showing its diabetogenic properties (Giongo et al., 2011; De Goffau et al., 2013; De Goffau et al., 2014; Leiva-Gea et al., 2018). In agreement with prior studies, the abundance of the Bacteroidetes phylum was observed to significantly increase the risk of T1D in our study. In addition, Bacteroidia class and Bacteroidales order, both of which were subcategories of the Bacteroidetes phylum, were also observed to have a causal relation with T1D risk. The influence of the Firmicutes phylum on T1D conveyed an exactly inverse pattern with respect to the Bacteroidetes phylum in existing studies (Giongo et al., 2011; Leiva-Gea et al., 2018). Although the causal relation of the Firmicutes phylum and T1D was null in our study, we identified a significant subcategory, *Eubacterium eligens* group genus, which showed a protective effect on T1D. A recent literature performed an in vitro experiment and revealed that *Eubacterium eligens* and its culture supernatant strongly promoted the production of anti-inflammatory IL-10 by epithelial cells, suggesting its potential to confer anti-inflammatory activity in vivo and deliver health benefits (Chung et al., 2017). In addition to four significant estimates mentioned above, other nominally significant estimates, with IVW-derived *P* value < 0.05 and no detected heterogeneity and pleiotropy, should also be treated cautiously. Future studies are expected to further profile compositional changes in gut microbiota in T1D cases and dissect the role of specific bacterial taxa in the pathophysiology of T1D.

Overgrowth of some microorganisms and loss of others lead to an imbalance of the gut microbial ecosystem and a followed loss of important physiological functions, which is defined as gut microbiome dysbiosis (Knip and Siljander, 2016; Mokhtari et al., 2021). Dysbiosis has been documented in T1D pathogenesis (Harbison et al., 2019). Aberrant gut microbiota composition might play a pivotal role in the progression and development of T1D mainly *via* modulating the formation of short-chain fatty acids (SCFAs), altering intestinal permeability, and regulating immune and inflammatory response. Previous experiments reported a rapid disease onset and high T1D incidence in non-obese diabetic (NOD) mice under germ-free conditions, indicating that the existence of diabetes-protective commensal microbes (Wen et al., 2008; Tlaskalová-Hogenová et al., 2011). Butyrate, one of the representatives of SCFAs and mainly produced by Firmicutes, possesses anti-inflammatory properties and enhances the intestinal barrier by up-regulating the tight-junction (TJ) protein (Mills et al., 2019). A newly published research demonstrated that oral administration of butyrate exerted antidiabetic effects in the T1D mouse model through promoting the serum C-peptide level, alleviating the islet lesions, and increasing numbers of islets and total insulin-positive islets (Yuan et al., 2022). Also, the abundance of butyrate-producing bacteria was reversely associated with the number of β-cell autoantibodies within the body, as evidenced by a population study (De Goffau et al., 2013). Therefore, the decreased fraction of butyrate-producing bacteria in the gastrointestinal tract might participate in the pathogenesis of T1D. Some maleficent bacteria and their metabolic products can regulate the assembly of tight junctions (TJs) to influence gut permeability (Mokhtari et al., 2021). Increased intestinal permeability has been observed in both diabetes-prone individuals and animal models in comparison with their controls (Neu et al., 2005; Bosi et al., 2006; Maffeis et al., 2016). This alteration allows the transit of luminal contents like dietary antigens, exogenous antigens, and microbial components to the underlying tissues and further into the bloodstream, causing the activation of the immune system and the promotion of a state of inflammation (Viggiano et al., 2015). A newly published research proved that breakage of gut barrier continuity can lead to activation of islet-specific T cells within the intestinal mucosa and to autoimmune diabetes using the preclinical mouse model (Sorini et al., 2019). All these findings came together to indicate that the increased gut permeability that occurred before the onset of the clinical disease is related to T1D pathogenesis rather than a secondary alteration induced by T1D. Two recent experimental studies simulated gut microbiome dysbiosis by feeding NOD mice with antibiotic in early life and observed enhanced T1D incidence paralleled by altered expressions of genes controlling both innate and adaptive immunity and abnormalities of innate immunity and T-cell differentiation (Livanos et al., 2016; Zhang et al., 2018). As Siljander concluded from published animal studies, prolonged or repetitive deviation from the optimal microbial homeostasis (dysbiosis) may lead to loss of self-tolerance and spreading of proinflammatory signals and effector cells (Siljander et al., 2019).

This study has vital clinical significance. Since specific bacterial taxa implicated in T1D risk had been detected, it provided the possibility for researchers to seek for innovative interventions aimed at the prevention and treatment of T1D through focusing on human gut microbiota as a breaking point. Notably, oral probiotics/prebiotics seems a prospective means. So far, emerging evidence had documented that oral supplementation of probiotics/prebiotics was capable of preventing diabetes development in NOD mice and prediabetic individuals (Calcinaro et al., 2005; Dolpady et al., 2016; Zhang et al., 2022). In our study, the *Eubacterium eligens* group genus was the only taxa identified to have a significantly protective effect on T1D, and its anti-inflammatory property had been proved in an in vitro experiment lately (Chung et al., 2017). This discovery is expected to be applied in the research and development of new probiotics/prebiotics to benefit “at-risk” individuals. Furthermore, fecal microbiota transplant (FMT) seems another promising approach. Antibiotic-treated mice transplanted with gut microbiota from T1D children showed significantly elevated fasting glucose levels and declined insulin sensitivity versus controls (Yuan et al., 2022). Conversely, colonization of germ-free NOD mice with the normal gut microbiota from human beings attenuates the process of T1D (Wen et al., 2008). Our study revealed several bacterial taxa which had a causal relation with T1D including Bacteroidetes phylum, Bacteroidia class, Bacteroidales order, and *Eubacterium eligens* group genus. This finding is conducive to identifying an optimal gut microbiota signature, paving the way for the feasibility of FMT as a means to attenuate even reverse disease progression for diabetic and prediabetic individuals.

Also, there are limitations. Firstly, the microbial taxa-related GWAS data totally included 18,340 participants from multiple ethnics, whereas the GWAS summary statistic data of T1D only included the Europeans. This issue had been considered carefully. We finally decided performed MR analysis using the two GWASs owing to the following three points: a. the Europeans accounted for the majority (nearly 80%) in the former GWAS data; b. it possesses the largest sample size and most bacterial taxa among currently available gut microbiota-related GWAS data; c. it had been used for MR analysis in convincing studies. Secondly, the original study on gut microbiota only meta-analyzed microbial taxa at five levels, namely, phylum, class, order, family, and genus, and consequently lacked GWAS summary statistics at the species level. Therefore, we failed to determine taxa at the species level causally related with T1D, which helps identify an optimal gut microbiota more accurately. Thirdly, the nominally significant taxa identified in our study should be treated dialectically. The *P* values all derived from the IVW method were less than 0.05, although they did not pass the BH correction. Thus, future studies are warranted to validate or exclude their role in T1D.

## 5 Conclusion

In conclusion, the study comprehensively analyzes the causal relation of genetically predicted gut microbiota at different levels and T1D using the two-sample bidirectional MR method. Our estimates reveal that Bacteroidetes phylum, Bacteroidia class, and Bacteroidales order causally increase T1D risk, whereas the *Eubacterium eligens* group genus, which belongs to the Firmicutes phylum, causally decreases T1D risk. The findings enable innovative interventions such as oral administration of probiotics/prebiotics and FMT as a means to restore a healthy microbiota, thereby reducing T1D risk. Nevertheless, future studies are warranted to further profile an optimal gut microbiota composition and dissect the underlying mechanisms of specific bacterial taxa’s role in the pathophysiology of T1D.

## Data availability statement

The datasets presented in this study can be found in online repositories. The names of the repository/repositories and accession number(s) can be found in the article/**Supplementary Material**.

## Author contributions

ML designed the study and wrote the first draft of the manuscript. ML, XL, QC, TZ, and JW gathered data and conducted statistical analyses. MS, TW, SZ, and XS participated in data interpretation. JQ and ML revised the manuscript for intellectual content. All authors read and approved the final manuscript.
